# Assessment of apathy in neurological patients using the Apathy Motivation Index caregiver version

**DOI:** 10.1111/jnp.12262

**Published:** 2021-09-16

**Authors:** Verena S. Klar, Yuen‐Siang Ang, Patricia Lockwood, Bahaaeddin Attaallah, Shannon Dickson, Daniel Drew, Annika Kienast, Maria R. Maio, Olivia Plant, Elitsa Slavkova, Sofia Toniolo, Rhea Zambellas, Sarosh R. Irani, Masud Husain

**Affiliations:** ^1^ Department of Experimental Psychology University of Oxford UK; ^2^ Social and Cognitive Computing Department Institute of High Performance Computing A*STAR Singapore Singapore; ^3^ Centre for Human Brain Health School of Psychology University of Birmingham UK; ^4^ Nuffield Department of Clinical Neurosciences University of Oxford UK; ^5^ Department of Neurology John Radcliffe Hospital Oxford University Hospitals UK; ^6^ Oxford Autoimmune Neurology Group Nuffield Department of Clinical Neurosciences University of Oxford UK; ^7^ Wellcome Trust Centre for Integrative Neuroimaging Department of Experimental Psychology University of Oxford UK

**Keywords:** apathy, motivation, neuropsychiatry, Alzheimer’s disease, Parkinson’s disease, Limbic encephalitis, Subjective cognitive impairment

## Abstract

Apathy is a common, disabling neuropsychiatric syndrome that occurs across many brain disorders and may be associated with diminished motivation in behavioural, cognitive, emotional and social domains. Assessment is complicated by the variability of symptoms across apathy domains and self‐report from patients, which can be misleading due to their lack of insight. Independent evaluation by clinicians also has limitations though if it has to be performed with limited time. Caregiver reports are a viable alternative, but current assessments for them either do not distinguish between different apathy domains or are interview‐based and take long to administer. In this study, we developed a brief caregiver questionnaire version of the recently developed Apathy Motivation Index (AMI), which is a self‐report tool. We confirmed three apathy factors in this new caregiver measure (AMI‐CG) that were also present in the AMI: Behavioural Activation, Emotional Sensitivity and Social Motivation. Furthermore, we validated the scores against more extensive caregiver interviews using the established Lillle apathy rating scale as well as patient self‐reports of apathy, measures of depression, anhedonia, cognition, activities of daily living and caregiver burden across four different neurological conditions: Parkinson's disease, Alzheimer's disease, subjective cognitive impairment and limbic encephalitis. The AMI‐CG showed good internal reliability, external validity and diagnostic accuracy. It also uncovered cases of social apathy overlooked by traditional instruments. Crucially, patients who under‐rated their apathy compared to informants were more likely to have difficulties performing everyday activities and to be a greater burden to caregivers. The findings provide evidence for a multidimensional conceptualization of apathy and an instrument for efficient detection of apathy based on caregiver reports for use in clinical practice.

## Background

Apathy is increasingly recognized to be a common, disabling syndrome characterized by impairments of motivation and associated with poor prognosis (Husain & Roiser, 2018; Starkstein & Leentjens, 2008). It is now considered a major neuropsychiatric manifestation of many brain disorders, including neurodegenerative and neuroinflammatory conditions, both common and rare. For example in Parkinson’s disease (PD), reported prevalence ranges from 17 to 70%, depending on assessment tools and comorbid symptoms (Brok et al., 2015), and higher apathy is predictive of cognitive decline over time (Martin, McDonald, Allsop, Diggle, & Leroi, 2020). In Alzheimer’s disease (AD), apathy is the most commonly observed and earliest behavioural change, present in 49% of patients, on the basis of pooled prevalence data (Zhao et al., 2016). In neuroinflammatory disorders such as multiple sclerosis apathy is present in ~40% of cases (Raimo, Spitaleri, Trojano, & Santangelo, 2020), while in a form of auotimmune encephalitis known as Anti‐LGI1 limbic encephalitis (LE), it has been documented in 53% of individuals (van Sonderen et al., 2016).

Additionally, it is increasingly recognized that apathy is prevalent amongst people at risk of developing dementia, such as those with mild cognitive impairment (MCI: with cognitive impairment apparent on cognitive screening) or subjective cognitive impairment (SCI: with subjective complaints but no apparent impairment on cognitive screening). SCI can precede MCI, which in turn can progress to AD (Jessen et al., 2014, 2020; Reisberg et al., 2008; Slot et al., 2018). In these groups, the reported prevalence ranges from 2.2 to 75%, with apathy being associated with a two‐fold increased risk of dementia (van Dalen et al., 2018).

Two important issues have emerged from investigations of apathy. The first concerns whether there are different, dissociable dimensions of the syndrome and how best to capture these when assessing a patient. The second, related issue is how best to measure apathy. Should the assessment rely on self‐report by the patient, the evaluation of a person who knows them well, such as a caregiver, or on independent interview of either the patient or the caregiver? All of these different types of assessment have been used with instruments that seek to dissociate different dimensions of the syndrome.

Apathy has been considered to have several different dimensions or dissociable domains. However, there is no consensus on how many domains there might be. Marin et al.’s triadic theory proposed three different axes of apathy: diminished productivity (behavioural apathy), diminished goals (cognitive apathy) and diminished emotional responses (affective apathy; Marin, Biedrzycki, & Firinciogullari, 1991). However, the Apathy Evaluation Scale (AES), developed to measure these hypothesized domains, instead supported three factors that the authors described as general apathy, curiosity or novelty seeking, and a third factor that contained items on insight, need for help with planning and lack of concern for problems (Marin et al., 1991).

Subsequent scales also tried to measure different dimensions of apathy, finding evidence for cognitive and behavioural (Pedersen et al., 2012; Starkstein, Petracca, Chemerinski, & Kremer, 2001) as well as emotional aspects of apathy (Robert et al., 2002). Other influential approaches have reframed the components or suggested additional ones. For example the Dimensional Apathy Scale (Radakovic & Abrahams, 2014) recovered four factors: executive, emotional, behavioural initiation and cognitive initiation while the Lille Apathy Rating Scale (LARS, Sockeel et al., 2006), reported four distinct factors including a new component of self‐awareness in PD. Finally, the Apathy Motivation Index (AMI) demonstrated behavioural and emotional factors, and a new factor that could best be described as social apathy which has been confirmed in healthy people and Parkinson’s disease (Ang, Lockwood, Apps, Muhammed, & Husain, 2017; Ang et al., 2018).

The variability of symptoms, across different domains and measured by different scales, renders assessing apathy a challenge. Moreover, there is no absolute ground truth as to whether a patient suffers from apathy and in which subdomain it manifests. Rather, the evidence suggests that this varies depending upon who reports on the patient’s symptoms. For example Clarke et al. (2007) examined apathy in dementia including patients with AD and dementia with Lewy bodies using three different versions of the AES and found two factors for the self‐report (general and other) and two factors using the caregiver and clinician versions (general and interest).

Several apathy scales now have different versions that allow patient, caregiver or clinician perspectives. Each of these has its drawbacks, but at the same time may highlight important details that the others fail to detect. For example, a shortcoming of the patient report is that they may have become habituated to their apathy or may lack the necessary insight or awareness, particularly relevant in populations with cognitive impairment. While clinician ratings based on patient interviews might be better, they are dependent on the patient’s cooperation – as well as insight and memory – and take time as well as trained personnel. In everyday clinical experience, many clinicians effectively rely on taking a history from a caregiver or informant, so this might seem a viable alternative to relying on self‐report, provided the caregiver report is reliable.

However, current formal assessment using instruments to assess and quantify caregiver ratings either fail to capture the range of apathy domains or take a long time to administer. One of the most widely used assessments in clinical settings is the Neuropsychiatric Inventory (NPI) (Cummings et al., 1994) which has a brief clinical version, the Neuropsychiatric Inventory Questionnaire (NPI‐Q; Kaufer et al., 2000). The informant‐based interview assessment screens for neuropsychiatric symptoms including apathy, but does not distinguish between apathy subtypes and attempts to divide its questions into domains have failed to detect a factor structure in frontotemporal dementia and Alzheimer's disease (Chow et al., 2009). Moreover, if the caregiver responds with a negative response to the screening question for apathy, further detailed questioning is not pursued, or scored, thereby risking false negatives. The informant version of the LARS (LARS‐i), on the other hand, consists of four distinct factors (Dujardin, Sockeel, Delliaux, Destée, & Defebvre, 2008), but the assessment is interview‐based and takes at least 15 min to administer. Furthermore, it does not provide an assessment of social apathy which was found to be a separate domain (Ang et al., 2018) and is recognized in revised diagnostic criteria for apathy (Robert et al., 2018).

A brief but detailed caregiver assessment of apathy that is clinically practical and also provides sufficient information on different domains of apathy, including the social domain, is currently not available. Here, we present an investigation of a caregiver version of the AMI (AMI‐CG). As in the original self‐report AMI, the questions attempt to distinguish between behavioural, emotional and social dimensions of apathy (Ang et al., 2018). Our aim was to provide a sensitive caregiver questionnaire that would assess these dimensions, but in contrast to interview‐based assessments, take less than 5 min to complete by a caregiver without independent clinician input and time. Apathy and related constructs, such as anhedonia and depression, were assessed in a sample of patients with diverse neurological conditions. The factorial structure was determined and internal reliability and external validity established. Then, we assessed whether it would provide sufficient diagnostic accuracy using the LARS‐i as gold standard. Finally, we explored whether discrepancies between self‐report and caregiver report are related to the patient’s cognitive ability or caregiver burden.

## Methods

### Participants

One hundred and thirty‐four patients with four different diagnosed neurological conditions and their caregivers were recruited from Neurology clinics participating in this study: AD (*N* = 28), Parkinson’s disease (PD, *N* = 48), SCI (*N* = 28) and autoimmune LE (*N* = 30; LGI1 or Caspr2 cases; Table 1). Of caregivers, 110 were spouses or partners, seven children, six siblings or other family members, seven friends and four not otherwise specified. In order to be included as a caregiver in the study, the participant needed to know the patient well enough to inform us about the impact of their condition. We deemed this given when they were either a spouse or partner (82% of the sample) or had known the patient for at least 3 years. Caregivers knew patients for an average of 39 years (*SD* = 15.7 years, see group‐wise statistics in Table 1). In cases where the caregiver was the spouse, they were of the opposite gender and a similar age. All participants, including caregivers, gave written informed consent; the study was approved by a local NHS ethics committee (REC number 18/SC/0448).

**Table 1:** Participant demographics

Diagnosis	Gender (F:M)^a^	Age (years)^b^	Age range	Education (years)^c^	ACE	Length of patient‐caregiver relationship
AD (*n* = 28)	13:13	72.78 ± 9.18	53–89	15.58 ± 5.4	71.93 ± 12.8	45.25 ± 11.22
LE (*n* = 30)	7:23	67 ± 9.66	47–87	12.38 ± 2.73	90.07 ± 7.98	38.45 ± 15.13
PD (*n* = 48)	18:27	70.13 ± 7.52	50–87	15.11 ± 3.47	91.78 ± 7.95	41.17 ± 16.17
SCI (*n* = 28)	14:14	58.29 ± 9.11	34–81	15.09 ± 4.09	92.04 ± 7.37	29.33 ± 15.48
Total (*n* = 134)	52:77	67.25 ± 10.06	34–89	14.49 ± 4.09	87.13 ± 12.13	39.02 ± 15.67

Note

AD, Alzheimer's disease; LE, Limbic encephalitis; PD, Parkinson's disease; SCI, Subjective cognitive impairment; ACE, Addenbrooke's Cognitive Examination III

^a^ *n* = 5 missing.

^b^ *n* = 6 missing.

^c^ *n* = 31 missing.

### Measures

Patients completed:

*Apathy‐Motivation Index (AMI)* (Ang et al., 2017; *N* = 134). This 18‐item self‐report questionnaire assesses apathy in terms of Behavioural Activation (tendency to self‐initiate goal‐directed behaviour), Social Motivation (level of engagement in social interactions) and Emotional Sensitivity (affective responses) using a 5‐point Likert scale. Item scores are averaged to yield scores for subscales and a total score with higher scores indicating greater apathy (range 0–4).

*Snaith–Hamilton Pleasure Scale (SHAPS)* (Snaith et al., 1995; *N* = 133). This self‐report questionnaire assesses hedonic tone, that is, the degree to which a person is able to experience or anticipate pleasure. It covers four domains: interest/pastimes, social interaction, sensory experience and food/drink, using 14 items and a four‐point scale (strongly disagree = 1/disagree = 2/agree = 3/definitely agree = 4). We used the original dichotomous scoring (agree = 1/disagree = 0) to determine which patients could be classified as anhedonic and a four‐point scoring system for more dispersion in the data (following Franken, Rassin, & Muris, 2007). Higher scores indicate greater anhedonia.

*Geriatric Depression Scale Short Form (GDS‐15)* (Yesavage & Sheikh, 1986; *N* = 133). This 15‐item, two‐point scale self‐report screening assesses depressive symptoms in older adults with the exception of somatic symptoms, providing a more robust measure in people with medical illness (yes/no, scores range 0–15 with higher scores indicated more severe depression).

*Beck Depression Inventory II (BDI‐II)* (Beck, Ward, Mendelson, Mock, & Erbaugh, 1961; *N* = 134). The longstanding self‐report inventory describes the varying degrees of depression using a four‐point scale with higher scores indicating more severe depression (Scores range 0–63).

*Addenbrooke’s Cognitive Examination‐III (ACE‐III)* (*N* = 128). This screening to assess cognitive functioning in five domains: attention, memory, verbal fluency, language and visuospatial skills. Scores range from 0 to 100 with lower scores indicating higher cognitive impairment.

Caregivers reported on patients’ apathy using:

*Apathy‐Motivation Index caregiver version (AMI‐CG)* (*N* = 134). Our new questionnaire developed using the original AMI, covers apathy in terms of Behavioural Activation, Social Motivation and Emotional Sensitivity domains. Table 3 shows items. Item scores are averaged to yield scores for subscales and a total score with higher scores indicating greater apathy (range 0–4).

*Lille Apathy Rating Scale caregiver version (LARS‐i)* (Dujardin et al., 2008; *N* = 129). The interview‐based caregiver version of LARS assesses apathy in four domains: Intellectual curiosity, emotion, action initiation, self‐awareness. Total scores range from −36 to 36 (least apathetic to most apathetic).

*Neuropsychiatric Inventory Questionnaire (NPI‐Q)* (Kaufer et al., 2000; *N* = 130). This interview screens for symptoms of psychopathology common in dementia, including apathy. It includes one screening question followed by eight yes/no questions if the screening is answered with yes and does not distinguish between domains.

Caregivers also reported on:

*Bayer Activities of Daily Living Scale (B‐ADL)* (Hindmarch, Lehfeld, Jongh, & Erzigkeit, 1998; *N* = 133). This questionnaire assesses patients’ deficits in performance of everyday activities, such as taking medication or using transportation using 25 items and a 10‐point response scale. Item scores are averaged to yield a total score ranging from 0 to 10 with higher scores indicating greater difficulty completing everyday tasks independently.

*Zarit Burden Interview (ZBI)* (Zarit, Todd, & Zarit, 1986; *N* = 133). This questionnaire assesses the burden caregivers experience from caring for their relative in 22 items using a 5‐point response scale Scores range from 0 to 88 with higher scores indicating greater caregiver burden.

### Statistical analyses

For the main analyses, data from patients and caregivers were collapsed across patient groups and analysed using R v3.6.1 (R Core Team, 2019). Correlational analyses used pairwise Spearman correlations and corrected for multiple inference using Holm’s method (Holm, 1979). Exploratory factor analysis and reliability analyses were conducted using R packages *psych* (Revelle, 2018) and *paran* (Dinno, 2018).

## Results

### Prevalence rates of cognitive impairment, apathy, depression and anhedonia

**Figure 1:**
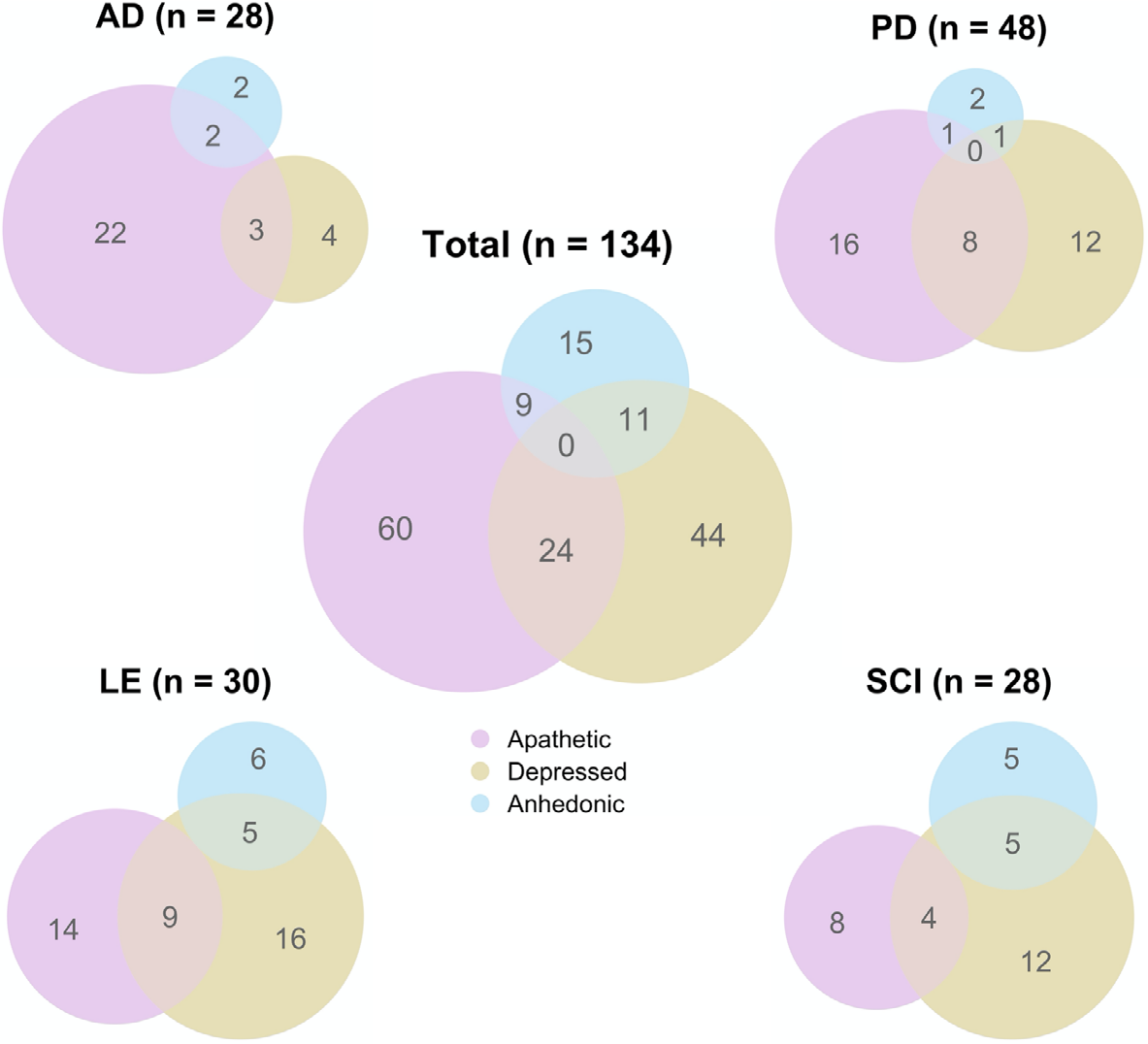
Overlap between neuropsychiatric symptoms. Circles show absolute numbers of cases of apathy, depression and anhedonia as well as the intersection of cases by patient group. AD, Alzheimer's disease; LE, Limbic encephalitis; PD, Parkinson's disease; SCI, Subjective cognitive impairment.

In order to assess prevalence rates of apathy and relevant neuropsychiatric features in our patient groups, we classified participants according to standard cut‐off values. Patients were classified as apathetic if *either* the AMI or the LARS‐CG indicated apathy, as depressed if either the BDI *or* the GDS indicated depression, as anhedonic based on the SHAPS, and cognitively impaired if the ACE. Absolute values and overlap between symptoms can be found in Figure 1; cut‐off values and prevalence rates are reported in Table 2. The AD group showed the highest rates of apathy (78.57%), followed by LE (46.28%), PD (34.78%) and SCI (32%). Similarly, the AD group showed the highest overlap between apathy and cognitive impairment (67.86), likely due to the high prevalence of cognitive impairment (89.3%). The highest overlap between apathy with depression (30.8%) and with anhedonia (15.4%) was found in LE.

**Table 2:** Prevalence of neuropsychiatric symptoms by patient group

	AD (*n* = 28)	LE (*n* = 30)	PD (*n* = 48)	SCI (*n* = 28)
Impaired^a^	25/28 (89.29%)	10/27 (37.04%)	11/45 (24.44%)	5/28 (17.86%)
Apathetic^b^	22/28 (78.57%)	14/29 (48.28%)	16/46 (34.78%)	8/25 (32%)
Depressed^d^	4/28 (14.29%)	17/29 (58.62%)	13/47 (27.66%)	15/28 (53.57%)
Anhedonic^d^	2/28 (7.14%)	6/30 (20%)	2/47 (4.26%)	6/28 (21.43%)
Apathetic & Impaired	19/28 (67.86%)	6/26 (23.08%)	8/41 (19.51%)	2/25 (8%)
Apathetic & Depressed	3/28 (10.71%)	8/26 (30.77%)	8/41 (19.51%)	4/25 (16%)
Apathetic & Anhedonic	2/28 (7.14%)	4/26 (15.38%)	1/41 (2.44%)	1/25 (4%)

Note

AD, Alzheimer's disease; LE, Limbic encephalitis; PD, Parkinson's disease; SCI, Subjective cognitive impairment.

^a^ Cognitive impairment indicated by a score smaller than 88 on the ACE. Missing data: 3 LE, 3 PD.

^b^ Apathy, indicated either by a score greater than 1.91 on the AMI (moderate apathy) or by a score greater than −16 on the LARS‐i. Missing observations: 1 LE, 2 PD, 3 SCI.

^c^ Depression, indicated either by a score greater than 17 (moderate depression) or by a score greater than 5 on the GDS. Missing data: 1 LE, 1 PD.

^d^ Anhedonia, indicated by a score greater than 2 on the SHAPS. Missing data: 1 PD.

### Factorial structures are similar for AMI Caregiver and AMI

Since the items of the AMI‐CG were adapted from the AMI, we expected them to map onto similar subscales of Behavioural Activation, Emotional Sensitivity and Social Motivation. In order to assess the factorial structure of the AMI‐CG, an exploratory factor analysis was first conducted. The Kaiser–Meyer–Olkin Test (Kaiser, 1974) which measures the proportion of shared variance among the data, indicated sampling was adequate for factor analysis (KMO = 0.82). Horn’s Parallel Analysis (Horn, 1965) for component retention determined that three factors should be retained based on 2,000 iterations. Thus, we conducted an exploratory factor analysis with three factors and Promax rotation, allowing factors to be correlated.

**Figure 2:**
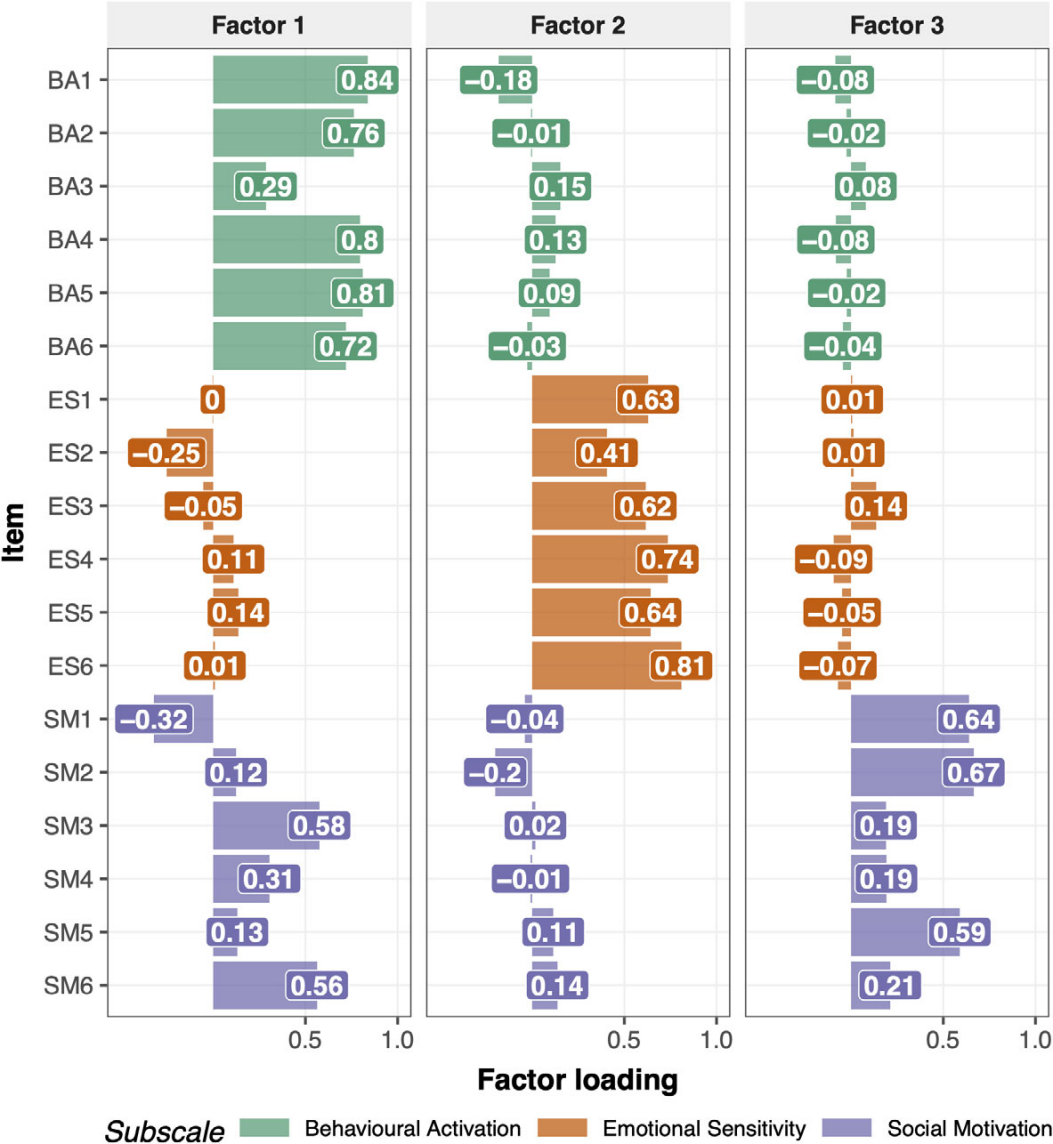
Factor structure of AMI‐CG. Results from the exploratory factor analysis of AMI‐CG items. Factors 1, 2 and 3 predominantly load on items from subscales Behavioural Activation (BA), Emotional Sensitivity (ES) and Social Motivation (SM) respectively. This shows that the AMI‐CG reproduces the initial structure of the AMI.

Results indicated that three factors were sufficient (*χ* ^2^ (102) = 170.26, *p* < .001), cumulatively explaining 46% variance. This structure had a good model fit (RMSEA = 0.077 with 90% CI of 0.052–0.089, RSMR = 0.05, TLI = 0.88). Furthermore, the factor structure of the original AMI was confirmed in the AMI‐CG, with Factor 1, 2 and 3 loading on all items of subscales Behavioural Activation, Emotional Sensitivity and Social Motivation respectively (mean absolute loadings 0.7, 0.64, 0.42). Additionally, Factor 1 had moderately high loadings on items of the Social Motivation subscale (mean absolute loading 0.34; Figure 2). Moreover, factors were intercorrelated, with Factor 2 (associated with Emotional Sensitivity) showing smaller correlations with the other factors (*r*_Factor3/Social Motivation_ = 0.42, *r*_Factor1/Behavioural Activation_ = 0.39, *p*‐values < .01) than Factor 3 (predominantly high Social Motivation items) and Factor 1 (predominantly high on Behavioural Activation items; *r* = .47, *p* < .01). These findings speak for a social domain of apathy that shares elements with aspects of behavioural apathy.

### AMI‐CG shows good reliability and construct validity across subscales

**Figure 3:**
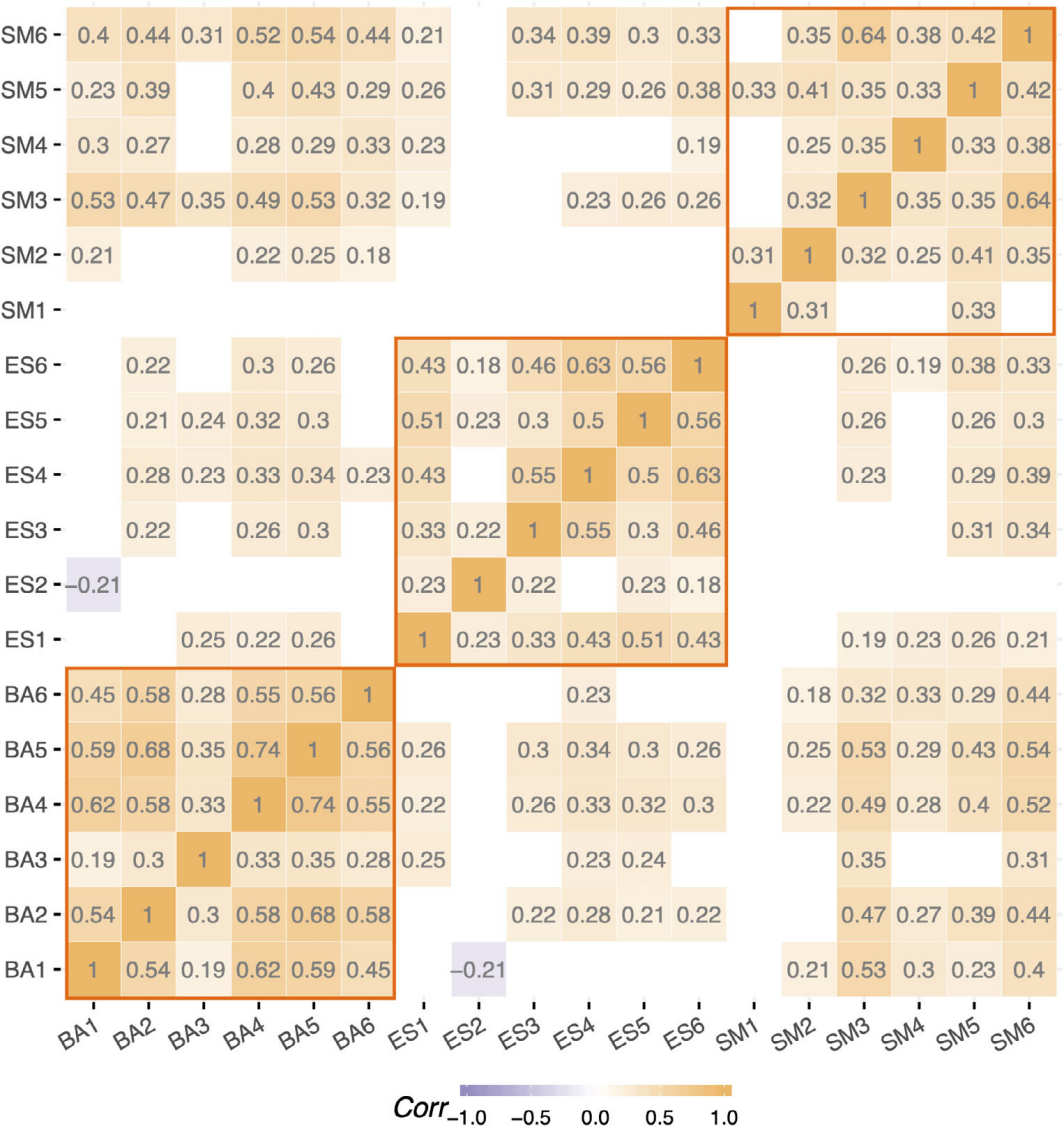
Correlations between AMI‐CG items ordered by subscale. Correlation matrix showing correlations between AMI‐CG items. Blank tiles indicate that the correlation did not reach significance at a significance level of *p* = .05. Orange lines highlight correlations between items within one subscale to illustrate internal consistency. BA, Behavioural Activation; ES, Emotional Sensitivity; SM, Social Motivation. *p < .05, **p < .01.

Next, we investigated reliability and construct validity of the new scale. Cronbach’s alpha values for AMI‐CG total scores and subscales demonstrated good internal reliability (α_overall_ = 0.85). Consistency across subscales ranged from good (α_Behavioural Activation_ = 0.85) to acceptable (α_Emotional Sensitivity_ = 0.79, α_Social Motivation_ = 0.70), providing evidence of reasonable reliability of the AMI‐CG. Figure 3 shows the pairwise item correlations of the AMI‐CG illustrating that items moderate‐to‐high correlations between items from the same subscale. Moreover, it shows also found low‐to‐moderate correlations between items from the Behavioural Activation and Social Motivation subscales (.18 ≤ *r* ≤ .53) and between Emotional Sensitivity and Social Motivation (.19 ≤ *r* ≤ .39), as well as low correlations between the Behavioural Activation and Emotional Sensitivity (−.21 ≤ *r* ≤ .33). Moreover, subscale scores correlated highly with the total score (*r* = .63–.84, *p*‐values < .01, Table 3).

**Table 3:** Apathy motivation index caregiver version item scores

Subscale	Item	Statement	Mean (*SD*)
Behavioural activation (BA)	BA1	Makes decisions firmly and without hesitation	1.91 (1.22)
	BA2	When he/she decides to do something, he/she is able to make an effort easily	1.43 (1.25)
	BA3	Doesn't like to laze around	1.84 (1.37)
	BA4	Gets things done when they need to be done, without requiring reminders from others	2.04 (1.28)
	BA5	When he/she decides to do something, he/she is motivated to see it through to the end	1.34 (1.18)
	BA6	When he/she has something they need to do, he/she can do it straightaway	1.61 (1.1)
Emotional sensitivity (ES)	ES1	Feels sad or upset when they hear bad news	0.91 (0.95)
	ES2	After making a decision, will wonder if they made the wrong choice	2.27 (1.04)
	ES3	Seems to care deeply about what their loved ones think of them	1.18 (1.1)
	ES4	Feels awful if they say something insensitive	1.69 (1.17)
	ES5	Feels bad when they hear an acquaintance has an accident or illness	0.8 (0.9)
	ES6	Feels guilty if he/she realizes he/she has been unpleasant to someone	1.26 (1.08)
Social motivation (SM)	SM1	Starts conversations with random people	2.15 (1.42)
	SM2	Seems to enjoy doing things with people he/she has just met	2.03 (1.12)
	SM3	Suggests activities to do	1.99 (1.26)
	SM4	Goes out with friends on a weekly basis	2.25 (1.51)
	SM5	Starts conversations without being prompted	1.42 (1.11)
	SM6	Enjoys choosing what to do from a range of activities	1.76 (1.17)

**Figure 4:**
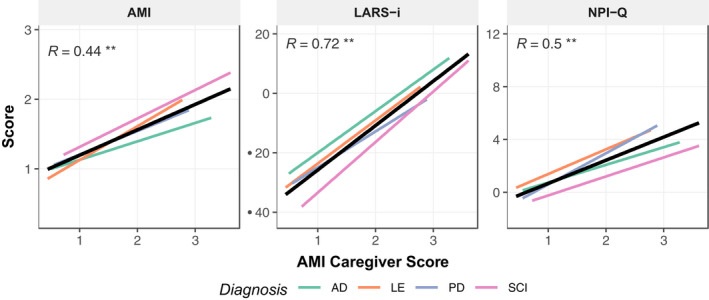
Correlation of AMI‐CG with other apathy measures by patient group. There was a moderate significant correlation between the AMI‐CG and the original AMI, a strong significant correlation between AMI‐CG and LARS‐i and a moderate relationship between the AMI‐CG and the NPI‐Q. AD, Alzheimer's disease; LE, Limbic encephalitis; PD, Parkinson's disease; SCI, Subjective cognitive impairment. *p < .05, **p < .01.

In order to assess construct validity, we examined correlations of all collected measures collapsed across patient groups (Table 4). The AMI‐CG total score demonstrated good convergent construct validity, correlating with other measures of apathy. It showed a strong correlation with LARS‐i total scores (*r* = .72, *p* < .01) and moderate correlations with NPI‐Q apathy score (*r* = .5, *p* < .01, Figure 4) and the AMI itself (*r* = .44, *p* < .01, Figure 4).

**Table 4:** Correlations between AMI caregiver total score and subscale score with related measures

	AMI‐CG total	Behavioural activation	Emotional sensitivity	Social motivation
Apathy measures				
Apathy Motivation Index Total (AMI)	0.44**	0.31**	0.26**	0.49**
Behavioural Activation	0.31**	0.33**	0.13	0.25**
Emotional Sensitivity	0.30**	0.14	0.32**	0.27**
Social Motivation	0.39**	0.24**	0.19*	0.51**
Apathy Motivation Index Caregiver Version Total (AMI‐CG)	–	0.84**	0.63**	0.81**
Behavioural Activation	0.84**	–	0.29**	0.55**
Emotional Sensitivity	0.63**	0.29**	–	0.34**
Social Motivation	0.81**	0.55**	0.34**	–
Lille Apathy Rating Scale Caregiver Version (LARS‐i)	0.72**	0.68**	0.44**	0.51**
Neuropsychiatric Inventory Apathy Score (NPI‐Q)	0.50**	0.49**	0.29**	0.33**
Related neuropsychiatric measures				
Beck's Depression Inventory (BDI)	0.18*	0.26**	−0.04	0.19*
Geriatric Depression Scale (GDS)	0.1	0.17	−0.08	0.18*
Snaith–Hamilton Anhedonia Scale (SHAPS)	0.27**	0.16	0.15	0.33**
Related caregiver measures				
Bayer Activities of Daily Living (B‐ADL)	0.52**	0.67**	0.13	0.32**
Zarit Burden Interview (ZBI)	0.56**	0.60**	0.31**	0.35**

Note

*p < .05, **p < .01.

**Figure 5:**
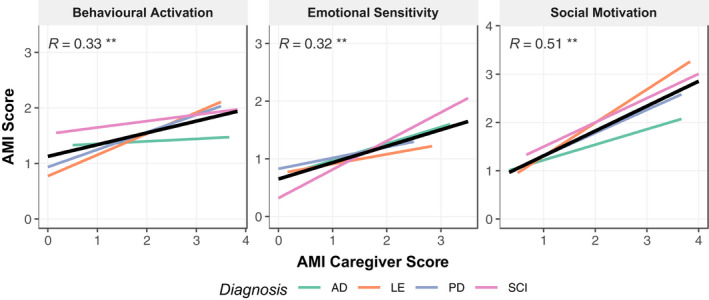
Correlation between AMI‐CG and AMI subscale scores by patient group. Low correlations between the AMI‐CG and AMI Behavioural Activation subscale. Low and moderate correlations between items of the Emotional Sensitivity subscale and moderate. Correlations between subscales did not hold for all patient groups (see text). AD, Alzheimer's disease; LE, Limbic encephalitis; PD, Parkinson's disease; SCI, Subjective cognitive impairment. *p < .05, **p < .01.

There was also considerable agreement between the subscales of the AMI and AMI‐CG, with the strongest relationship evident in the social domain (*r*_Social Motivation_ = 0.51, *p* < .01; *r*_Emotional Sensitiivty_ = 0.32, *p* < .01; *r*_Behavioural Activation_ = 0.33, *p* < .01; Figure 5).

Importantly, the total AMI‐CG score did not correlate with GDS (*r* = .1, *p* = .23) and only weakly with the BDI (*r* = .18, *p* = .04). Thus, apathy, as indexed by the AMI‐CG, was only weakly associated with established measures of depression, showing good discriminant construct validity. However, the AMI‐CG total scores did correlate moderately with the SHAPS index of anhedonia (*r* = .27, *p* < .01), perhaps consistent with recognition that some elements of anhedonia and apathy might overlap (Husain & Roiser, 2018). Summary scores of related measures are given in Table 5.

**Table 5:** Summary of relevant measures

	*M* (*SD*)	Median	Range
Apathy measures			
Apathy Motivation Index (AMI)	1.44 (0.51)	1.44	[0.28; 2.83]
Behavioural Activation	1.49 (0.71)	1.42	[0; 3.33]
Emotional Sensitivity	1.03 (0.6)	1	[0; 3.17]
Social Motivation	1.79 (0.79)	1.83	[0; 3.83]
Apathy Motivation Index Caregiver Version (AMI‐CG)	1.66 (0.65)	1.61	[0.44; 3.61]
Behavioural Activation	1.7 (0.93)	1.5	[0; 3.83]
Emotional Sensitivity	1.35 (0.73)	1.25	[0; 3.5]
Social Motivation	1.93 (0.81)	1.83	[0.33; 4]
Lille Apathy Rating Scale Caregiver Version (LARS‐i)	−15.96 (13.25)	−18.5	[−35; 20]
Neuropsychiatric Inventory Apathy Score (NPI)	1.84 (2.67)	0	[0; 12]
Related neuropsychiatric measures			
Beck's Depression Inventory (BDI)	11.57 (9.05)	10	[0; 45]
Geriatric Depression Scale (GDS)	4.05 (3.7)	3	[0; 15]
Snaith–Hamilton Anhedonia Scale (SHAPS)	21.83 (5.42)	22	[14; 38]
Related caregiver measures			
Bayer Activities of Daily Living (B‐ADL)	3.62 (2.25)	3.08	[1; 9.29]
Zarit Burden Interview	23.08 (16.14)	20	[0; 66]

Overall, correlations of related measures with the AMI‐CG subscale scores were similar to the correlations with the total score. Both the Emotional Sensitivity subscale, and the Social Motivation subscale showed lower albeit still significant correlations than the total score with the LARS‐i (*r* = .44 and *r* = .51, *p* < .01) and the NPI‐Q (*r* = .29 and *r* = .33, *p* < .05), suggesting that emotional and social apathy contributes to a lesser extent to the total scores of the LARS‐i and the NPI‐Q than behavioural apathy. Furthermore, unlike the total AMI‐CG Score, the Emotional Sensitivity subscale was the only one that did not correlate with either the BDI (*r* = −.04, *p* = .64), nor the GDS (*r* = −.08, *p* = .37), suggesting emotional apathy might be different from depression. Finally, while also weakly correlating with measures of depression (BDI, *r* = .18, *p* < .05; GDS, *r* = .19, *p* < .05), Behavioural Activation was the only subscale that correlated significantly with the SHAPS (*r* = .33, *p* < .01), suggesting that the relationship between anhedonia and apathy might be largely driven by social apathy. Correlations split up by disease group can be found in Table S1.

### AMI‐CG shows good diagnostic accuracy

**Figure 6:**
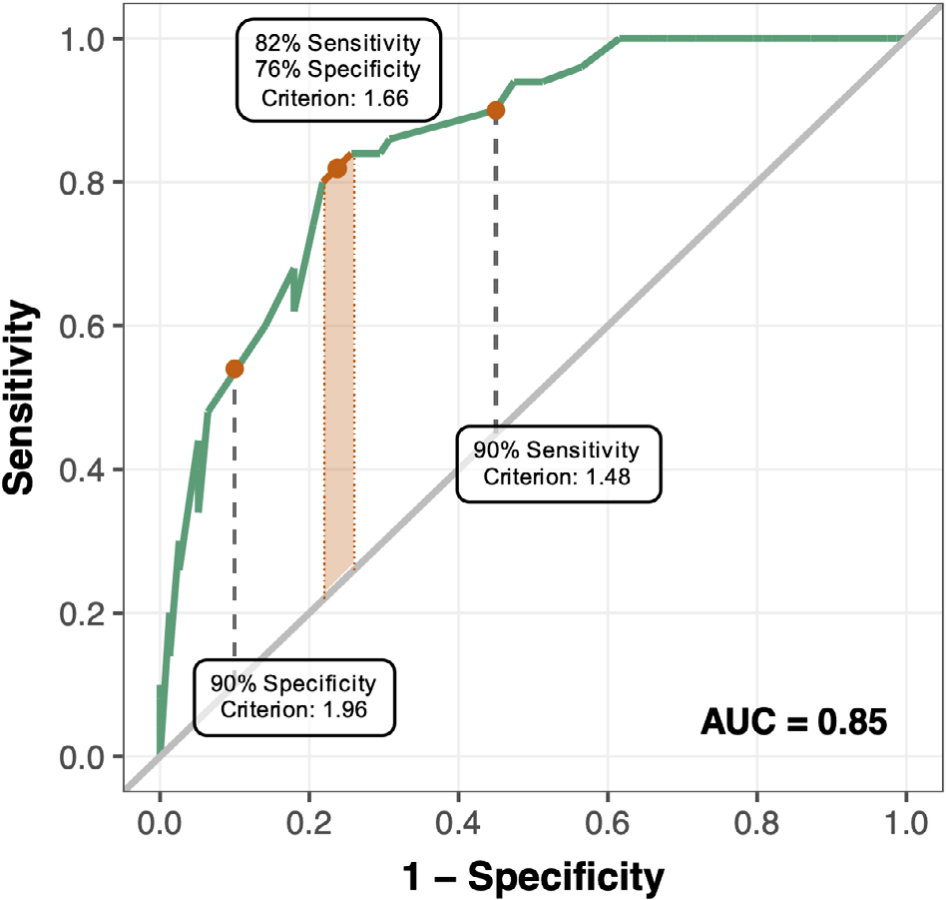
ROC curve showing Sensitivity and Specificity for different cut‐off criteria. ROC analysis performed by adopting LARS‐i as gold standard to determine cut‐off criterion demonstrated AMI‐CG has good diagnostic accuracy for clinical apathy (sensitivity = 0.82, specificity = 0.76, area under the curve (AUC) = 0.85). Optimal cut‐off is an AMI‐CG score of 1.66: patients with a score greater than this should be classified as apathetic. Using this threshold, the AMI‐CG correctly distinguishes between apathetic and non‐apathetic cases in 85% of cases. Two other cut‐off scores are provided: 1.96 for a 90% specific test (55% sensitive), prioritizing true negatives and 1.48 for a 90% sensitive test (55% specific), prioritizing true positives.

Using the LARS‐i with a cut‐off value of −16 as gold standard, we plotted the receiver operating characteristic (ROC, Figure 6) curve to determine the optimal cut‐off across 100 possible thresholds. Using Youden’s J statistic (Youden, 1950), two optimized criterion values for apathy at AMI‐CG scores of 1.64 and 1.68 were identified, resulting in an averaged optimal cut‐off value of score of 1.66 (*J* = 0.54), which is the value corresponding to the highest accuracy. Sensitivity (correct detection of apathy cases) and specificity (correct rejection of non‐apathy cases) for this threshold are 82% and 76% respectively. In addition, we identified cut‐off values leading to 90% sensitivity (cut‐off 1.48) and 90% specificity (cut‐off 1.96) for circumstances in which one measure may be of greater importance. Sensitivity and specificity across candidate thresholds can be found in Table 6. Using this criterion, 42 and 58 individuals, respectively, were correctly identified as apathetic and non‐apathetic. The area under the curve was 0.85, meaning that the threshold would be able to distinguish between apathetic and non‐apathetic patients in 85% of cases (Figure 6).

**Table 6:** Criterion‐related validity to find an optimized criterion for the AMI‐CG

Criterion	Sensitivity	Specificity	Youden's J
**0.00**	1.00	0.00	0.00
**0.40**	1.00	0.00	0.00
**0.80**	1.00	0.17	0.17
**1.20**	1.00	0.38	0.38
**1.48** ^a^	**0.90**	**0.54**	**0.44**
**1.66** ^b^	**0.82**	**0.76**	**0.54**
**1.96** ^c^	**0.55**	**0.90**	**0.45**
**2.00**	0.51	0.92	0.43
**2.40**	0.31	0.97	0.28
**2.80**	0.10	1.00	0.10
**3.20**	0.06	1.00	0.06
**3.60**	0.02	1.00	0.02

^a^ AMI‐CG cut‐off for 90% sensitivity.

^b^ AMI‐CG cut‐off for 90% specificity.

^c^ Optimal AMI‐CG according to Youden's J statistic, resulting in 82% sensitivity and 76% specificity.

### AMI‐CG detects social apathy

While this criterion gives acceptable sensitivity of 82%, specificity is moderate at 76%. In our sample, the AMI‐CG and LARS‐i classified 62 and 50 patients as apathetic, respectively, corresponding to 48.4% and 39% of the sample. In order to understand the source of the low specificity, we examined the ‘false positives’, that is, the cases identified by the AMI‐CG but not by the LARS‐i. The 20 patients classified as non‐apathetic by LARS‐i and as apathetic by the AMI‐CG scored high predominantly on items in the Social Motivation domain of the AMI‐CG (*M*_Behavioural Activation_ = 1.97, *M*_Emotional Sensitiivty_ = 1.63, *M*_Social Motivation_ = 2.43). In particular, they scored highly (were rated apathetic) on the basis of caregiver responses to two items: *Starts conversations with random people* (SM1, *M *= 2.85, *SD* = 1.14) and *Goes out with friends on a weekly basis* (SM4, *M* = 3.1, *SD* = 1.02). In contrast, patients classified as non‐apathetic according to NPI‐Q did not show systematically higher ratings on any of the AMI‐CG subscales. Thus, the AMI‐CG appears to detect social apathy, which is not a separate domain in the LARS‐i. This appears to explain why the AMI‐CG detected more cases of apathy than the LARS‐i.

### Discrepancy between caregiver and patient reports related to caregiver burden and cognitive deficits

Overall, AMI‐CG total scores (*M* = 1.66, *SD* = 0.65) were significantly higher than AMI total scores (*M* = 1.44, *SD* = 0.51) (*t*(238.91) = −3.13, *p* = .002), even though, as previously discussed, AMI‐CG total score was positively correlated with the original AMI (Figure 4) and subscales of the two questionnaires were correlated (Figure 5). Thus, these analyses reveal that while there is some commonality to patient and caregiver reports, there are also differences. The perspective – from that of the caregiver or from the point of view of the patient – when assessing a patient’s apathy matters significantly.

**Figure 7:**
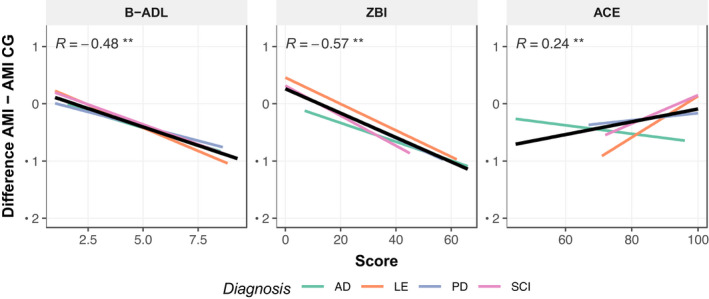
Relationship between discrepancy in self‐ and caregiver report by patient group. There were significant correlations between the difference in self‐report (AMI) and caregiver report (AMI‐CG) scores and performance of everyday activities (B‐ADL), Caregiver Burden (ZBI) and measures of cognitive ability (ACE). AMI, Apathy Motivation Index; AMI‐CG, AMI Caregiver; B‐ADL, Bayer's Activities of Daily Living; ZBI, Zarit Burden Interview; ACE, Addenbrooke's Cognitive Examination III; AD, Alzheimer's disease; LE, Limbic encephalitis; PD, Parkinson's disease; SCI, Subjective cognitive impairment. *p < .05, **p < .01.

In order to understand what drives the discrepancy, we examined the difference in AMI – AMI‐CG scores, that is, the extent to which caregivers rated patient’s apathy lower than the patient themselves. These difference scores correlated significantly negatively with Bayer Activities of Daily Living (*r* = −.48, *p* < .01; and a trend in the AD group, *r* = −.35, *p* < .07) and Zarit Burden Interview scores (*r* = −.57, *p* < .01). Conversely, they correlated significantly positively with ACE‐III cognitive scores (*r* = .24, *p* < .01; Figure 7), although this was driven by a strong correlation in the LE group (*r* = .49, *p* < .01), that did not reach significance in the other groups (0.44 ≤ *p* ≤ .49). In other words, the greater a caregiver rated a patient’s apathy compared to the patient themself, the worse the patient’s functional independence and the greater the overall burden to the caregiver.

This suggests that individuals who are more severely affected by their illness, with respect to their functional ability, and those who are the biggest burden to caregivers actually have the strongest discrepancy, with patients evaluating their apathy to be far less than their caregiver. However, B‐ADL scores themselves correlated moderately with ACE‐III (*r* = −.48, *p* < .01) and ZBI scores (*r* = .69, *p* < .01), and there was a significant relationship between ACE‐III and ZBI (*r* = −.25, *p* < .01). Future research in much larger sample sizes, might consider more advanced analyses to identify mediating roles of these variables.

## Discussion

In this study, we validated the AMI caregiver version (AMI‐CG), a new questionnaire that can be completed rapidly by someone who knows a patient well, to provide ratings of apathy independent of the patient. The investigation was performed across a diverse range of brain disorders, given that the syndrome of apathy is a major neuropsychiatric manifestation that occurs commonly across many diseases. The AMI‐CG showed good reliability. Exploratory factor analysis revealed a similar factorial structure to the original AMI (which relies on self‐report), identifying the same subscales, namely Behavioural Activation, Emotional Sensitivity and Social motivation. The AMI‐CG also showed good convergent and divergent external validity. It demonstrated a strong relationship with other measures of apathy (Figure 4), but no significant correlation with established indices of depression and only a weak correlation with anhedonia. Measured against an established, comprehensive caregiver interview for apathy symptoms, the LARS‐i, the AMI‐CG showed good diagnostic accuracy. In addition, it detected cases of social apathy that were missed by the LARS‐i. Finally, discrepancies between patient rating (AMI) and caregiver rating (AMI‐CG) were moderately related to caregiver burden and weakly related to the patient’s cognitive deficits (Figure 7).

### Evidence for multidimensionality of apathy, including a social dimension

In line with previous studies, the current factor analysis provided further evidence for apathy as a multidimensional construct. The analysis performed on this data set clearly favoured a structure with three factors: Behavioural Activation, Social Motivation and Emotional Sensitivity (Figure 2). Correlations between subscales indicated a larger overlap for Social Motivation and Behavioural Activation, whereas Emotional Sensitivity seemed more distinct.

Crucially, the analysis provided conceptual support for a dimension of social apathy. Furthermore, patient and caregiver reports corresponded best in the Social Motivation domain, potentially because behavioural changes in this domain are best defined or more noticeable in everyday life. Thus, this domain may be helpful for diagnostic purposes, as proposed by new diagnostic criteria for apathy (Robert et al., 2018).

Despite the good sensitivity and overall diagnostic accuracy scores of the AMI‐CG, the specificity was comparatively low (0.76; see Figure 6). One explanation for this is that most patients classified as non‐apathetic on the LARS‐I but apathetic by the AMI‐CG scored high on social apathy. Since a social domain is not covered by the LARS‐i, which we used as a gold standard, this accounts for the apparent low specificity. It may be an important strength of the AMI‐CG that it can detect social apathy which was also established as a distinct factor of apathy using the self‐report AMI questionnaire (Ang et al., 2017).

One issue that might be of some concern is that neither the AMI nor the AMI‐CG specifically distinguish between behavioural and cognitive domains of apathy. This is not to say that such separate domains do not exist. Rather, it is often the case that neither patients themselves nor their caregivers can easily distinguish between these different dimensions. What is either acknowledged (by a patient) or observed (by a caregiver) is a lack of activity in everyday life. Some of this paucity of behaviour might indeed be due to lack of intellectual curiosity (perhaps captured by the term ‘cognitive apathy’) but the outward manifestation is often in terms of lack of activity (behaviour). Although deeper clinical interviewing might tease these aspects apart, the spontaneous reports of patients and caregivers might not easily do so. Therefore, measures of behavioural apathy might potentially be subsuming aspects of cognitive apathy which are acknowledged in new diagnostic criteria (Robert et al., 2018).

### Caregiver reports provide additional perspective

One important issue in apathy research has concerned whether the assessment should rely on self‐report from the patient, evaluation of a person who knows them well such as a caregiver, or on an independent interview of either the patient or the caregiver by a clinician. All of these different types of assessment have been used previously but, as discussed in the Introduction, each has its own potential shortcomings and limitations. Here, we chose to examine caregiver report without input from a clinician, a method that potentially saves time and means that the AMI‐CG might also be used as a rapid screening tool, which could be followed‐up with more detailed questioning, if required.

The analyses performed here revealed that although the AMI‐CG scores (provided by caregivers) and AMI scores (self‐reported by patients) were significantly correlated, there were also significant differences (Figure 4). Overall, caregivers rated apathy higher than the patient themselves, which might not be surprising. Further analysis showed that the discrepancy between self‐reported AMI and caregiver AMI‐CG scores was weakly related to patient cognitive ability and moderately to caregiver burden (Figure 7). While our data do not support causal claims, this adds to our understanding of the rating discrepancy.

One explanation is that the more cognitively impaired the patient is, the less insight they have into their apathy, leading to less accurate ratings. Such anosognosia would suggest an *underestimation by the patient* rather than an overestimate by the caregiver. Indeed, several studies have observed this effect and used it to measure awareness. Seltzer and Brennan (2001) found that both Alzheimer’s and Parkinson’s disease patients rated their apathy lower than their caregivers. Using the Apathy Inventory ratings from caregiver and patient versions, Robert et al. (2002) found that awareness of lack of interest, emotional blunting and lack of initiative, were all impaired in Alzheimer's disease. However, although these studies equated the discrepancy with lack of awareness of the patient, they cannot rule out alternative explanations. For example anosognosia as measured by the rating discrepancy has been related to overall more severe cognitive impairment in AD (Seltzer & Brennan, 2001). Our data are in line with these findings, showing that overall caregivers rated apathy higher the more cognitively impaired the patient was (Figure 7). In summary, these studies suggest that higher caregiver ratings compared to patient ratings can result from a lack of insight, but further research could explore this relationship using alternative measures of awareness.

The findings presented here also offer another explanation for the discrepancy. Caregivers tended to rate apathy higher when the patient was more dependent on them for daily tasks or they experienced high burden from caring for them. This replicates previous findings that showed caregiver bias in several domains, including apathy, depression and quality of life to be related to caregiver burden (Pfeifer, Horn, Maercker, & Forstmeier, 2017). The generality of the bias suggests that as caregivers become more burdened, this factor might negatively influence their perception of a patient's symptoms. However, neither Pfeifer et al.’s study nor our study can rule out a domain‐general underestimation or a form of anosognosia in patient evaluations. Moreover, the connection between cognitive impairment and caregiver burden has been found to be mediated by awareness as measured by rating discrepancy (Seltzer, Vasterling, Yoder, & Thompson, 1997), suggesting that these explanations are not necessarily mutually exclusive.

Overall, the findings presented here demonstrate that given the nature of questionnaires, it is difficult to establish a ground truth on the severity of apathy. Relying solely on patient self‐reports might be misleading, including for the evaluation of therapeutic interventions or clinical trials. The difference in patient versus caregiver evaluation might be a profitable area for future research on the impact of apathy on individual clinical outcomes, as well as on the development of behavioural measures of apathy.

### Limitations

The main limitations to this study are group differences in sample size, cognitive impairment and length of patient‐caregiver relationship. There were several differences between the patient groups. Sample sizes were limited, in particular in the AD, limbic encephalitis and SCI groups. These were half the size of the Parkinson's group, so our overall findings might be more generalizable to Parkinson's patients. Patients from the Alzheimer's, limbic encephalitis and Parkinson's group tended to be 10 years older than the SCI group, who as a result also showed shorter patient–caregiver relationships. Finally, the Alzheimer's group was on average more cognitively impaired than the other groups. While these differences should not impact the validation of our questionnaire, they could be confounding factors for some of the findings on discrepancy we did not control for. For example the caregiver’s age may influence how burdened they feel by caring for their relative, and cognitive impairment may influence the extent to which rating discrepancies can be explained by lack of insight.

As a final limitation, we would like to note that our criteria for including someone as a caregiver were based on the status or length of the relationship (spouse/partner). However, we could not provide precise data on the frequency of contact between patient and caregiver, which would be a more fine‐grained measure of closeness and a potential predictor for the discrepancy between patient and caregiver.

Following our analysis of the discrepancy between patient and caregiver ratings, we suggest that the AMI‐CG should be preferred over self‐reports in cases where the patient’s cognitive impairment may prevent an accurate diagnosis. However, it would be prudent to be aware of the possibility that in cases of high caregiver burden and/or cognitive impairment, the caregiver rating might overestimate the subjective apathy of the patient.

### Conclusion

The AMI‐CG is a compact, clinically practical instrument that does not rely on patient self‐report and is quick and easy to administer. It provides an assessment from a person who knows the patient well, thereby overcoming potential concerns that rapid assessments performed by a clinician interviewing a patient might not capture the true extent of apathy at home. The results presented here show that the AMI‐CG successfully detects apathy including cases of social apathy that are overlooked by other measures, and it is not confounded by symptoms of depression which frequently co‐exist in patients with apathy.

## Conflicts of interest

All authors declare no conflict of interest.

## Authors’ contributions

Y.A., P.L. and M.H. were involved in task conceptualization. V.K., B.A., S.D., D.D., A.K., M.M., O.P., E.S., R.Z. and S.I. were involved in task administration. V.K. and M.H. carried out analysis, planning and execution of the paper and also writing the original draft of the paper.

## Supporting information

Table S1 Correlations between AMI Caregiver Total Score with Related Measures by Disease Group

## Data Availability

The data that support the findings of this study are available from the corresponding author upon reasonable request.

## Apathy Motivation Index – caregiver version

Below are a number of statements. Each statement asks you to think about the person you are answering for on the basis of the last 2 weeks. For each statement, select how appropriately it describes the persons thoughts and behaviours. Select “Completely true” if the statement describes them perfectly, “Completely untrue” if the statement does not describe them at all, and use the answers in between accordingly.

**Table:**

Name of person you are answering for	__________________
Relationship to the person	__________________
Length of time you have known another	__________________

**Table:**

		Completely untrue	Mostly untrue	Neither true nor untrue	Quite true	Completely true
1	Feels sad or upset when they hear bad news	□	□	□	□	□
2	Starts conversations with random people	□	□	□	□	□
3	Seems to enjoy doing things with people he/she has just met	□	□	□	□	□
4	Suggests activities to do	□	□	□	□	□
5	Makes decisions firmly and without hesitation	□	□	□	□	□
6	After making a decision, will wonder if they made the wrong choice	□	□	□	□	□
7	Seems to care deeply about what their loved ones think of them	□	□	□	□	□
8	Goes out with friends on a weekly basis	□	□	□	□	□
9	When he/she decides to do something, he/she is able to make an effort easily	□	□	□	□	□
10	Doesn’t like to laze around	□	□	□	□	□
11	Gets things done when they need to be done, without requiring reminders from others	□	□	□	□	□
12	When he/she decides to do something, he/she is motivated to see it through to the end	□	□	□	□	□
13	Feels awful if they say something insensitive	□	□	□	□	□
14	Starts conversations without being prompted.	□	□	□	□	□
15	When he/she has something they need to do, he/she can do it straightaway	□	□	□	□	□
16	Feels bad when they hear an acquaintance has an accident or illness	□	□	□	□	□
17	Enjoys choosing what to do from a range of activities	□	□	□	□	□
18	Feels guilty if he/she realises he/she has been unpleasant to someone	□	□	□	□	□

## Scoring instructions

Each item is negatively scored, i.e., you will need to *reverse all items*:

Three domains of apathy‐motivation are assessed with the mean score, which ranges from 0–4 with 0 being motivated and 4 being apathetic.

**Table:**

**(1) Behavioural Activation**	Q5, 9, 10, 11, 12, 15
**(2) Social Motivation**	Q2, 3, 4, 8, 14, 17
**(3) Emotional Sensitivity**	Q1, 6, 7, 13, 16, 18
