# Supplementary material for: Assessment of apathy in neurological patients using the Apathy Motivation Index caregiver version
Source: J Neuropsychol. 2021 Sep 16;16(1):236–58. doi: 10.1111/jnp.12262 (PMC9290131; doi:10.1111/jnp.12262)
Supplement: Supplementary file 1 — Table S1 Correlations between AMI Caregiver Total Score with Related Measures by Disease Group [file JNP-16-236-s001.docx]

Supplementary Material

Consistency of results across disease groups

We examined the consistency between AMI and AMI-CG separately for each patient group. The results can be found in Supplementary Table 1. shows the correlations of the AMI total score with related neuropsychiatric and caregiver measures by patient group. With a few exceptions, correlation significance and magnitude were similar across patient groups. In particular, the strong correlations with the LARS-i (0.66 ≤ r ≤ 0.8) and moderate correlations with the NPI-Q (0.37 ≤ r ≤ 0.56) highlight that convergent validity holds across patient groups. As an exception to this, the correlation with the NPI-Q narrowly did not reach significance in the SCI group (r = 0.37, p = 0.7). For the related measures, we found that the correlation with depression was largely driven by the LE group (r = 0.39, p < 0.01), indicating a stonger relationship of apathy and depression in LE than in other groups. Furthermore, AD and PD patients seemed to be the only groups showing a significant relation of AMI-CG scores to anhedonia indexed by the SHAPS (r = 0.40, p < 0.05 and r = 0.38, p < 0.01), although there was a trend for LE patients too (r = 0.34, p = 0.07).

## Supplementary Table 1. Correlations between AMI Caregiver Total Score with Related Measures by Disease Group

|  | AD  (*n* = 28) | LE  (*n* = 30) | PD  (*n* = 48) | SCI  (*n* = 28) |
| --- | --- | --- | --- | --- |
| Apathy Measures |  |  |  |  |
| Apathy Motivation Index (AMI) | 0.39 * | 0.56 ** | 0.46 ** | 0.41 * |
| Lille Apathy Rating Scale Caregiver Version (LARS-i) | 0.69 ** | 0.69 ** | 0.66 ** | 0.80 ** |
| Neuropsychiatric Inventory Apathy Score (NPI-Q) | 0.40 * | 0.53 ** | 0.56 ** | 0.37 |
| Related Neuropsychiatric Measures |  |  |  |  |
| Beck's Depression Inventory (BDI) | 0.09 | 0.39 * | 0.18 | 0.02 |
| Geriatric Depression Scale (GDS) | -0.26 | 0.32 | 0.27 | 0.00 |
| Snaith-Hamilton Anhedonia Scale (SHAPS) | 0.40 * | 0.34 | 0.38 ** | 0.09 |
| Related Caregiver Measures |  |  |  |  |
| Bayer Activities of Daily Living (B-ADL) | 0.41 * | 0.74 ** | 0.42 ** | 0.39 * |
| Zarit Burden Interview | 0.52 ** | 0.65 ** | 0.58 ** | 0.47 * |

* p < 0.05, ** p < 0.01
